# THE ASSOCIATION OF FRAILTY AND MORTALITY IN OLDER ADULTS: A SYSTEMATIC REVIEW AND META-ANALYSIS OF COHORT STUDY

**DOI:** 10.1093/geroni/igad104.2797

**Published:** 2023-12-21

**Authors:** Xianqi Gao, Rhayun Song, Moonkyoung Park, Yuelin Li, Linyu Lyu, Eunna Oh, Xing Fan

**Affiliations:** Chungnam National University, Daejeon, Republic of Korea; Chungnam National University, Daejeon, Republic of Korea; Chungnam National University, Daejeon, Republic of Korea; Chungnam National University, Daejeon, Republic of Korea; Chungnam National University, Daejeon, Republic of Korea; Chungnam National University, Daejeon, Republic of Korea; Chungnam National University, Daejeon, Republic of Korea

## Abstract

Frailty is associated with poor health outcomes in later life, improving lifespan and death through different mechanisms. This study aimed to systematically review the epidemiological evidence from cohort studies investigating the association between frailty and all-cause mortality, and to determine if frailty affects mortality in older adults. We searched for studies published and indexed in 4 electronic databases (EMBASE, PubMed, Cochrane Library, and Web of Science) and manual search and identified 28 cohort studies before Dec 31, 2022. We include studies if their design was longitudinal; the exposure of interest was measured frailty; the endpoint of interest was mortality; they provided a risk estimate (i.e., hazard ratio [HR]) and the corresponding 95% CI for the association between frailty and mortality. They used different indexes as their frailty as exposed definitions. Two investigators independently screened the full-text articles for inclusion. We used a random-effects model to obtain pooled HRs. Thirty-one studies were included for qualitative review, of which twenty-eight were eligible for meta-analysis, and included 67,114 individuals from 16 countries. Compared with healthy adults, frail adults had a significantly higher mortality risk. The pooled HR for mortality per increment of 0.1 frailty of community-dwelling adults was 2.33 (95% CI 2.05-2.65; I2 76.9%). The present study demonstrated that frailty was not only significantly related to an increased mortality risk but was also a strong predictor of mortality in community-dwelling adults.

